# Trends in Antipsychotic Drug Use in the United States, 2000–2016

**DOI:** 10.3390/pharmacy14010014

**Published:** 2026-01-24

**Authors:** Nisrine Haddad, Nawal Farhat, Jennifer Go, Yue Chen, Christopher A. Gravel, Franco Momoli, Donald R. Mattison, Douglas McNair, Abdallah Alami, Daniel Krewski

**Affiliations:** 1 School of Epidemiology and Public Health, University of Ottawa, Ottawa, ON K1H 8M5, Canada; 2 School of Mathematics and Statistics, Carleton University, Ottawa, ON K1S 5B6, Canada; 3 Risk Sciences International, Ottawa, ON K1Z 7T1, Canada; 4 Department of Mathematics and Statistics, University of Ottawa, Ottawa, ON K1N 6N5, Canada; 5 Data Literacy Research Institute, University of Ottawa, Ottawa, ON K1N 6N5, Canada; 6 Arnold School of Public Health, University of South Carolina, Columbia, SC 29208, USA; 7 Bill and Melinda Gates Foundation, Seattle, WA 98109, USA; douglas.mcnair@gatesfoundation.org

**Keywords:** trends in antipsychotic drug use, first generation antipsychotic drugs, second generation antipsychotic drugs, off-label use, electronic health records, drug utilization, pharmacoepidemiology

## Abstract

This study evaluated long-term trends in the prevalence of use of atypical and typical antipsychotic drugs (APDs), both as classes of drugs and as individual drugs, among adult inpatients in the United States (US). The Health Facts^®^ database developed by Cerner Corporation was used to analyze the prevalence of APD use among adult inpatients aged 18 years or older who were administered at least one antipsychotic medication order during hospitalization between 1 January 2000 and 31 December 2016. The prevalence of APD use was standardized by age, sex, race, and census region. Typical and atypical antipsychotic treatment patterns in the US differed over this period. While the use of atypical APDs increased overall, the use of typical antipsychotic medications decreased, but remained more prevalent. Overall, haloperidol and prochlorperazine were the two most administered antipsychotic medications throughout the study period. From 2000 to 2011, prochlorperazine and haloperidol were the first- and second-most prescribed typical APDs, respectively; haloperidol became the most administered antipsychotic of this class as of 2012. Quetiapine was the most administered atypical antipsychotic medication, followed by risperidone and olanzapine until 2014, after which olanzapine was the second-most administered atypical APD. There was a notable decline in the use of atypical antipsychotics medications between 2005 and 2008, which may reflect the impact of the Food and Drug Administration’s warnings and the American Diabetes Association’s consensus position, but only for a short time. The usage patterns observed in this study support existing evidence of substantial off-label use of antipsychotic drugs in the US.

## 1. Introduction

Antipsychotic drugs (APDs) can be classified as typical or first-generation antipsychotics (FGAs) and atypical or second-generation antipsychotics (SGAs). The discovery of clozapine in the 1950s, the prototype of atypical APDs, led to the expansion and subsequent use of this class of drugs [1]. However, clozapine was only approved to treat treatment-resistant schizophrenia by the United States Food and Drug Administration (US FDA) in 1989 [2,3]. This is due to reports on clozapine-induced inflammation associated with rapid titration [4,5,6] and the increased risk of agranulocytosis [3,7], as well as ongoing efficacy and safety studies for the drug [3]. APDs are indicated to treat psychotic disorders, mainly schizophrenia and bipolar disorder [1,8,9]. They are also used off-label to treat a wide range of neurological and behavioral conditions, including treatment-resistant depression, obsessive–compulsive disorder, autism spectrum disorder, sleep disorders and agitation [1]. The FDA approved quetiapine to treat schizophrenia and bipolar disorder, and haloperidol to treat schizophrenia, Tourette syndrome, and certain behavioral disorders and hyperactivity in children [10].

There are important differences between these two classes of antipsychotic agents. At clinically effective doses, atypical antipsychotics cause less undesirable extrapyramidal effects, such as dystonia and tardive dyskinesia, than typical antipsychotics [1]. This clinical property makes them a more attractive treatment option than their predecessors. Secondly, the two classes may differ in their mechanisms of action. Typical APDs act mainly as antagonists on dopamine 2 (D2) receptors, while atypical APDs are antagonists to serotonergic neurotransmitters, such as 5-hydroxytryptamine receptor 2A (5-HT_2A)_ and have other non-D2 receptor-mediated actions [1,11,12,13].

Over the last two decades, it has become evident that APDs, although effective, may cause serious adverse events. Because of this, the US FDA issued an alert in 2005 to convey concerns that the use of atypical APDs can cause mortality in elderly patients suffering from dementia. A second alert was subsequently issued in 2008 extending this concern to typical antipsychotics. Other drug safety communications ensued, specifically targeting the increased risk of skin reactions or new impulse control problems associated with APDs [14,15,16] (Table 1).

In recent years, several studies assessed the use of APDs in patient populations suffering from major depressive disorder (MDD), Parkinson’s disease, and anxiety disorders among those treated in different care settings [17,18,19]. Despite safety concerns regarding APD use in older adults and an initial awareness of the risks associated with atypical APDs in those suffering with dementia [20,21], these agents continue to be used to treat dementia, schizophrenia, and other psychotic disorders in older adults, including long term treatment, despite safety concerns [22]. Furthermore, controlled trials have established benefits of atypical APDs to treat insomnia, obsessive–compulsive disorder, anxiety, and other conditions [23,24]. However, longitudinal studies are complicated by the characteristics of the underlying conditions and the side effects of these medications, which may prompt irregular use by patients.

There is also increasing evidence that APDs are associated with metabolic [25] and neurological and metabolic side effects [26]. In particular, atypical APDs have been associated with obesity, diabetes, and dyslipidemia [27,28], and may increase the risk of cardiovascular disease. This led the American Diabetes Association (ADA) to publish consensus positions on the risk of atypical APDs for obesity and diabetes, promoting regular monitoring for metabolic syndrome [27].

The objective of this study is to establish long-term trends in the prevalence of APD use by class, and by individual typical or atypical antipsychotics in the US. We hypothesized that as the prevalence of use of atypical APDs increased over the years, the use of typical APDs would decrease. We also sought to examine the possible effects of FDA warnings on antipsychotic medication use in the US population.

## 2. Materials and Methods

### 2.1. Data Source

Study data were derived from Cerner Corporations’ Health Facts^®^ database for the period between 1 January 2000 and 31 December 2016. This database captures and stores de-identified, longitudinal electronic health records (EHRs), and includes information on patient demographics, encounters, diagnoses, prescriptions, procedures, laboratory tests, location of services/patients, hospitals, and billing. Health Facts contains data from over 65 million patients with over 84 million acute admissions, emergency, and ambulatory visits, as well as more than 151 million orders for nearly 4500 drugs by name and brand. It was previously used in the analysis of trends in medication use among inpatients, including concomitant use of clopidogrel and proton pump inhibitors [29], and antiparkinsonian drugs [30].

### 2.2. Study Population

All encounters with patients 18 years of age or older who were hospitalized between 1 January 2000 and 31 December 2016 were identified for each quarter for each calendar year in the database. All inpatients meeting these criteria comprised the study cohort. To ensure that only patients with available pharmacy data in Health Facts were included in the study, eligible encounters had to have at least one recorded medication order [29]. We restricted the analysis to individuals with complete demographic information (age, sex, and race) to allow for direct standardization [30]. Unknown and missing data on age, sex and race were excluded. Demographic data that were considered including patient age (patient age was stratified into categories: 18–64 years, 65–84 years and ≥85 years), sex (male or female), and race (Caucasian, African American, Hispanic, or other). Care setting characteristics included census region (Northeast, South, Midwest, or West), urban or rural setting, and teaching status of the health care facility. Analyses were conducted cross-sectionally for each calendar quarter. Patients with multiple encounters could be included in multiple quarters but were only counted once within a given quarter.

### 2.3. Exposure Assessment

The quarterly and annual prevalence of APD use among adult inpatients who were administered at least one antipsychotic drug, extracted from inpatient pharmacy medication orders, was standardized by age, sex, race, and census region. APDs approved by the FDA were classified into two classes (Table 2).

While the recent literature classifies certain agents (e.g., aripiprazole, cariprazine) as third-generation antipsychotics due to their partial D2 receptor agonist activity [31,32,33], we have included them here within the broader second-generation category.

**Table 2:** List of and Year of Approval of Atypical (*n* = 13) and Typical (*n* = 12) Antipsychotic Drugs approved by the Food and Drug Administration in the United States.

Atypical APDs (Year of Approval)	Typical APDs (Year of Approval)
Aripiprazole (2002)	Chlorpromazine (1954)
Asenapine (2009)	Droperidol (1963)
Brexipiprazole (2015)	Fluphenazine (1961)
Caripiprazine (2015)	Haloperidol (1976)
Clozapine (1989)	Loxapine (1975)
Iloperidone (2009)	Molindone (1974)
Lurasidone (2010)	Perphenazine (1961)
Olanzapine (1996)	Pimozide (1985)
Paliperidone (2006)	Prochlorperazine (1956)
Pimavanserin (2016)	Thioridazine (1964)
Quetiapine (1997)	Thiothixene (1967)
Risperidone (1993)	Trifluoperazine (1963)
Ziprasidone (2001)	

US FDA: [34].

All drug orders that were dispensed were considered valid and assumed to have been used by the patient. Considering the nature of the conditions for which APDs are prescribed, we assumed they were administered to the patient by hospital staff as prescribed. The number of inpatients with an antipsychotic medication order was calculated quarterly and annually by drug class and for individual drugs from 1 January 2000 to 31 December 2016. Annual data were reported in the main manuscript, with quarterly information presented in Supplemental Materials (Tables S4–S6).

### 2.4. Statistical Analysis

The prevalence of antipsychotic use was analyzed cross-sectionally by year and quarter. The prevalence of use was defined as the ratio of the number of unique patients that received at least one antipsychotic drug during a hospital encounter to the total number of unique patients that met the inclusion criteria during the same period. Data from the 2010 American Community Survey (USA Census Bureau) were used to calculate annual prevalence of antipsychotic drug use to examine prescribing trends over time, as standardized by age, sex, race, and census region. To better understand patterns of antipsychotic use by class, we stratified our analysis into typical and atypical APDs. All statistical analysis were conducted using SAS 9.4 (SAS Institute Inc., Cary, NC, USA). Quarterly prevalence estimates for APD use are shown in Supplemental Materials (Tables S4–S6).

## 3. Results

### 3.1. Demographics, Geographic Distribution, and Care Setting Characteristics

A total of 5,539,077 adult inpatients with 8,551,028 distinct encounters that met the inclusion criteria were identified. The mean age of subjects was 51.3 years (SD = 19.6) with the majority of inpatients aged 18 to 64 years (64.8%). Females accounted for 59.8% of inpatients, while males accounted for 40.2%. Of patients with a known race, 75.4% were reported as Caucasian, 15.1% as African American and 2.0% as Hispanic, with 7.5% classified as other races. The Southern and Northeast census regions had the highest proportions of patients, accounting for approximately one-third of the population at 33.6%% and 31.5% each, while the Midwest and Western regions of the United States were represented by 18.4% and 16.6% of adult inpatients, respectively. Most patients were admitted to an urban hospital (80.3%), and 67.8% of healthcare organizations were teaching facilities (Table 3).

### 3.2. Prevalence of Use of Antipsychotic Drugs by Class

**Figure 1:**
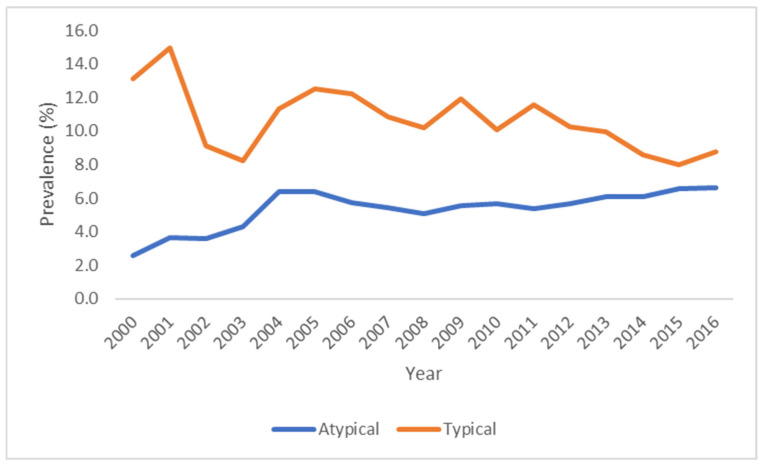
Standardized Annual Prevalence of Use of Antipsychotic Drugs by Class in Adult Inpatients, 2000–2016, United States.

The use of typical antipsychotics decreased during the study period while the use of atypical antipsychotics increased between 2000 and 2016 (Figure 1, Supplemental Materials Table S1). These trends support our hypothesis that as atypical APDs became widely used in recent years, the use of their predecessors decreased overall.

In 2000, the prevalence of use of atypical antipsychotics was 2.6% and fluctuated throughout the study period, increasing to 6.6% in 2016, representing a percent change of 153.6%. An evident increase in this class of agents was observed as of 2004, with slight fluctuations until the end of the study period. A noticeable decrease was observed between 2005 and 2008, which coincides with the FDA’s warnings pertaining to their use. Although the use of typical antipsychotics decreased from 13.1% in 2000 to 8.8% in 2016 (a 32.8% decrease), it is evident from this trend that these drugs continue to be widely used in the US. A decrease in typical APD was observed between 2005 and 2008 and after 2013, but this change was most noticeable between 2001 and 2002.

### 3.3. Prevalence of Use of Typical Antipsychotic Drugs

**Figure 2:**
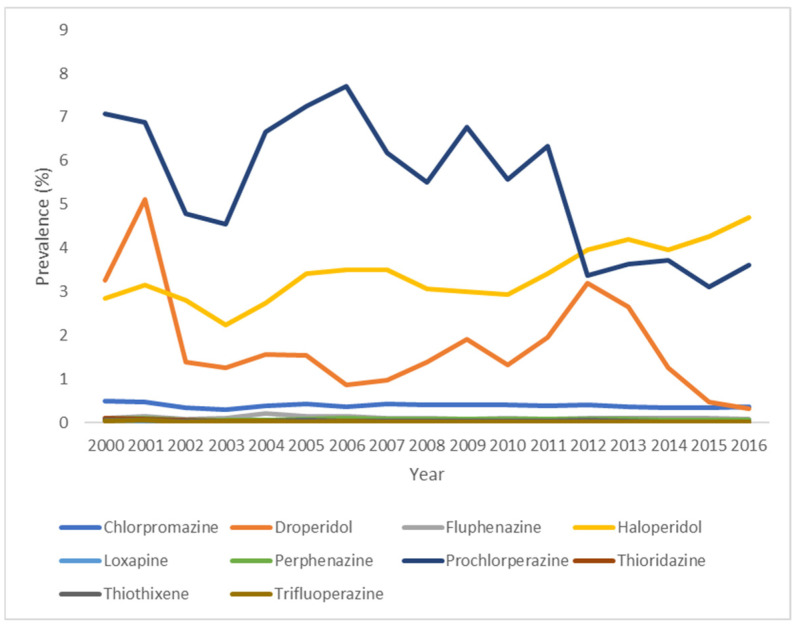
Standardized Annual Prevalence of Use of Individual Typical Antipsychotic Drugs in Adult Inpatients, 2000–2016, United States. Molindone and pimozide did not have any prevalence of use and are not included.

The annual use of individual typical APDs in the United States between 2000 and 2016 are shown in Figure 2 (Supplementary Materials Table S2). Prochlorperazine and haloperidol represented the first- and second-most administered typical antipsychotic medications until 2011, after which haloperidol became the most used drug of this class.

Although the prevalence of typical antipsychotics use fluctuated over the study period, an overall decrease was observed for most antipsychotics, except for haloperidol. There was a notable decrease in prevalence of use for chlorpromazine (decreasing from 0.5% in 2000 to 0.37% in 2016), droperidol (3.25% in 2000 and 0.33% in 2016), and prochlorperazine (7.08% in 2000 and 3.61% in 2016) across the study period, and a consistent decrease for all three drugs since 2012. In contrast, the use of haloperidol (2.85% in 2000 and 4.69% 2016) increased overall, while the use of fluphenazine (0.11% in 2000 and 0.09% in 2016) remained relatively stable but showed an overall decline. The prevalence of use of loxapine, perphenazine, thioridazine, thiothixene, and trifluoperazine consistently decreased.

### 3.4. Prevalence of Use of Atypical Antipsychotic Drugs

**Figure 3:**
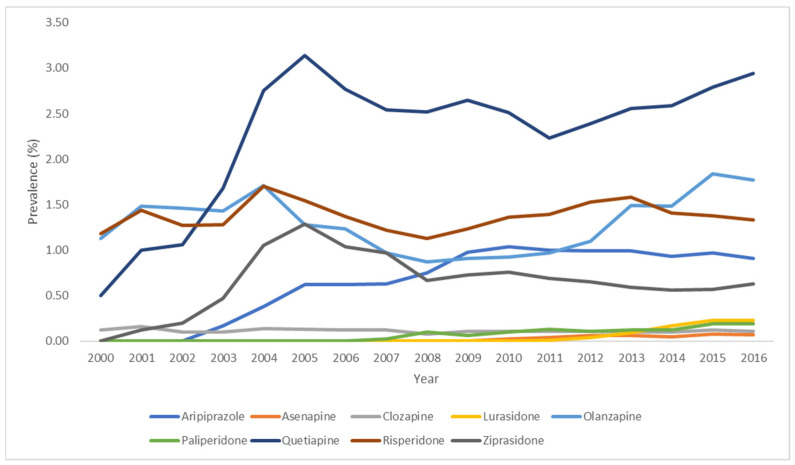
Standardized Annual Prevalence of Use of Individual Atypical Antipsychotic Drugs in Adult Inpatients, 2000–2016, United States. Brexipiprazole, caripiprazine and pimavanserin did not have any prevalence of use and are not included.

As shown in Figure 3 (Supplementary Materials Table S3), the prevalence of use of atypical APDs increased overall for individual drugs over the study period, with slight fluctuations. Quetiapine was the most administered drug (increasing from 0.50% in 2000 and 2.94 in 2016), followed by olanzapine (1.13% in 2000 and 1.77% in 2016), and risperidone (1.18% in 2000 and 1.33% in 2016). Risperidone was consistently more commonly used than olanzapine between 2005 and 2013, and olanzapine became the second-most prevalent atypical APD beginning in 2014. The prevalence of use of clozapine remained stable during the study period (0.12% in 2000 and 0.11% in 2016). Other drugs were not available in 2000, but their use increased following market authorization: ziprasidone prevalence increased from 0.12% in 2001 to 0.63% in 2016, and aripiprazole increased from 0.17% in 2003 to 0.91% in 2016.

The patterns of use of atypical APDs as a class demonstrated a notable decrease between 2005 and 2008. Individually, there was a marked decrease in the use of olanzapine and risperidone beginning in 2005, as well as for quetiapine and ziprasidone beginning in 2006, which coincides with the timing of FDA’s alerts and the ADA’s consensus position on atypical APDs. The use of aripiprazole, a newer drug, increased between 2003 and 2009, and then continued to be consistently used (Figure 3, Supplementary Materials Table S3).

## 4. Discussion

The present study examined trends in the use of APDs in the US from 2000 to 2016. Atypical APD use increased over the study period, while the use of typical APDs decreased during the same time. Quetiapine was the most administered atypical antipsychotic medication, followed by risperidone and olanzapine. From 2000 to 2011, prochlorperazine and haloperidol were the first- and second-most administered typical antipsychotic agents, respectively; haloperidol became the most administered typical APD as of 2012. Overall, haloperidol remained the most prescribed antipsychotic agent throughout the study period, followed by prochlorperazine.

A recent study by Su et al. (2020) [35] compared trends in antipsychotic medication use in Asia and the US. Findings from a 5% random sample form the United States Medicare Database showed that quetiapine had the highest incidence (new user) and prevalence rates in the United States (incidence rate (IR): 8.1–9.5 per 1000 patient-years, and prevalence rate (PR): 18.0–18.4 per 1000 subject, 2007–2011) in those aged 65 years of age or older. In patients younger than 65 years of age, quetiapine continued to have the highest incidence and prevalence rates, followed by risperidone (IR: 14.2–18.2 per 1000 patient-years; PR: 55.7–56.0 per 1000 subjects, 2007–2011). Weber et al. (2015) [36] examined data from 30 adult psychiatric inpatients who received an APD at The Ohio State University Wexner Medical Center. Quetiapine was the most prescribed atypical APD in inpatients suffering from anxiety disorders in 2013 (56.7%), followed by aripiprazole at 16.7%. Olanzapine and risperidone were both prescribed at a prevalence of 13.3%. In this study, the only typical antipsychotic prescribed to inpatients was haloperidol with a prevalence of 10%. Findings on outpatient settings are further described below.

Herzig et al. (2016) [18] evaluated patterns of APDs use in adult inpatients admitted to an urban academic medical center in Boston, Massachusetts, between August 2012 and August 2013 for conditions other than primary psychiatric disorders, including delirium, anxiety, and agitation. Nine percent of total adult admissions (*n* = 1537) were prescribed APDs, with 83% receiving atypical antipsychotics and 32% receiving typical antipsychotics. Fifteen percent of patients were exposed to both drug classes. Interestingly, 55% of inpatients taking APDs were started on this medication post-admission. A subgroup analysis by indication of use found that 53% and 12% of initiations of treatment were prescribed for delirium and probable delirium, respectively. In this analysis, quetiapine and olanzapine were the most prevalent atypical APDs at 3.3% each and haloperidol was the most commonly used typical APD at 2.4%, consistent with our findings that these drugs were among the most commonly used, although the prevalence of use in Health Facts differed. While we did not conduct similar subgroup analyses on indications for use, these findings suggest that APDs continue to be prescribed off-label to treat other conditions such as delirium. Haloperidol and atypical APDs are commonly used to treat this condition, especially in those experiencing severe illness [37]. However, the benefits of using haloperidol and atypical APDs, such as olanzapine, risperidone and quetiapine, to treat delirium are not always evident [38].

A more recent study analyzed the prescription patterns of APDs in adult inpatients admitted to a large community teaching hospital in Georgia between 2016 and 2017 [39]. Haloperidol was the most used antipsychotic, which corroborates our findings: the authors suggested this could be due to the possible administration of this drug by different routes, and could provide potentially more evidence-based indications for this antipsychotic. Ziprasidone, olanzapine, quetiapine, and risperidone were the most frequently used atypical APDs after haloperidol. In our analysis, the prevalence of use of ziprasidone was higher in 2016 compared to 2000, but decreased consistently as of 2005. The variations in prescribing patterns could be due to different prescribing practices in hospitals across the US as previously suggested [18]. This is also supported by additional evidence of unexplained variation in antipsychotic prescribing rates in nursing homes [40,41].

In our inpatient cohort, although we did not analyze the use of APDs by age, we observed a notable decline with APD treatment between 2005 and 2008. This trend aligns broadly with patterns reported in outpatient dementia populations following FDA warnings (Kale et al. 2011) in elderly patients with dementia [42]. Kale et al. 2012 [43] suggested quetiapine may be associated with a lower mortality risk relative to haloperidol in an elderly patient population with dementia after analyzing data from the US Department of Veteran Affairs, although APD use overall remains linked to adverse outcomes in this population. Haloperidol, one of the most studied and frequently used APDs, continues to be widely used worldwide. It is also effective in treating delirium due to fewer anticholinergic effect [44], which may partly explain its consistent use in the United States. Similarly, a review by Furik et al. (2015) [45] noted that prochlorperazine has been an effective and safe antiemetic to treat severe nausea when compared to other drugs such as droperidol, another typical APD (Din et al. 2023) [46].

In another study, Kim et al. (2018) [47] analyzed patterns of antipsychotic use in older adult inpatients (65 years or older) using data from the Premier Healthcare Database, which contains information from over 700 hospitals accounting for 20% of all hospitalizations in the US. The authors suggested that antipsychotic medications were often prescribed off-label for postoperative delirium. While the use of haloperidol and risperidone declined between 2004 and 2014, the use of quetiapine tripled over this same period.

Other studies focused on the prevalence of antipsychotic use in outpatient settings. While this is not within the scope of our analysis, differences in usage patterns may be attributed to how different care settings operate. Dennis and colleagues (2020) [48] reported that prevalence of combined antipsychotic use (atypical and typical) in US adults was 1.6% (*n* = 320) over a 5-year period from 2013 to 2018 using pooled data from the National Health and Nutrition Examination Survey (NHANES) of non-institutionalized residents. This is consistent with an estimated prevalence ranging from 0.88 to 1.73% in a previous outpatient setting analysis [49]. Aripiprazole and quetiapine accounted for 40.8% and 32.3% of prescriptions, followed by risperidone at 13.2%, while haloperidol and prochlorperazine were prescribed to 1.1% and 7.0% of outpatients, respectively, in an academic psychiatric setting in 2013 [36]. Rhee et al. (2018) [19] examined rates of APDs prescribing between 2006 and 2015 in adults with MDD in office-based outpatient settings. APD prescriptions initially increased between 2006 and 2009 (18.5% to 24.9%) and then decreased to 18.9% in 2015, and outpatient visits of patients aged 75 years or older declined significantly over the 10-year study period, reflecting FDA concerns about adverse events of antipsychotics in the elderly. Quetiapine, aripiprazole, and risperidone were the most prescribed atypical APDs at 36%, 27.7%, and 22%, respectively, throughout the study period. Haloperidol was prescribed to a significantly lesser extent, but remained the most commonly prescribed typical antipsychotic medicine. Bower et al. (2018) [17] measured the prevalence of patients with Parkinson’s disease taking antipsychotics in Olmsted County, Minnesota; of the 296 patients examined on 1 January 2006, the overall prevalence of antipsychotic use was 9.8%, with quetiapine being the most prevalent APD used on that day.

### 4.1. Strengths

This study evaluated the prevalence of APD use in a large inpatient cohort derived from multiple treatment centers, spanning a 16-year period and representing all four census regions of the US population. To our knowledge, this is the largest study exploring trends in APD use by class and by individual drugs. The results are standardized for age, sex, race, and census regions to reflect the demographics of the US population. Our analysis is consistent with findings from previous studies that were more limited in scope and population size.

### 4.2. Limitations

Our study is subject to several limitations. First, the study was restricted to inpatients who were administered at least one antipsychotic drug. As such, we could not draw direct comparisons between inpatient and outpatient settings. Second, we did not consider the concomitant use of other medications—other psychotropic drugs—which could influence the prescription of antipsychotics at the individual patient level. Lastly, there is a limited understanding of antipsychotic prescription practices within US hospitals, which could have been influenced by clinical preference, drug pricing, and availability.

## 5. Conclusions

As expected, typical and atypical antipsychotic prescribing patterns in the United States differed over the period between 2000 and 2016. There was a notable decline in the use of atypical antipsychotics between 2005 and 2007, which may be a direct impact of FDA warnings and the ADA’s consensus position, but only for a short time. Usage patterns observed in this study supports existing evidence of off-label use of antipsychotic drugs in the United States.

## Figures and Tables

**Table 1:** List of Alerts and Safety Communications Issued by the Food and Drug Administration Related to Antipsychotic Drugs ^1^.

Year	Type of Warning	Antipsychotic Medicine	Description
2005	FDA Alert	Atypical •Aripiprazole •Olanzapine •Olanzapine fluoxetine •Quetiapine •Risperidone •Clozapine •Ziprasidone	Increased mortality in the elderly
2008	FDA Alert	All APDs: Typical and atypical	Increased mortality in elderly patients with dementia-related psychosis
2011	Drug Safety Communication	All APDS: Typical and atypical	Drug labels updated on use during pregnancy and risk of abnormal muscle movements and withdrawal symptoms in newborns
2011	Drug Safety Communication	Atypical •Asenapine maleate	Serious allergic reactions
2014	Drug Safety Communication	Atypical •Ziprasidone	Associated with rare but potentially fatal skin reactions
2015	Drug Safety Communication	Atypical: •Clozapine	FDA modifies monitoring for neutropenia associated with schizophrenia medicine clozapine; approves new shared REMS program for all clozapine medicines
2016	Drug Safety Communication	Atypical •Aripiprazole	New impulse-control problems to gamble, binge eat, shop, and have sex
2016	Drug Safety Communication	Atypical •Olanzapine	Rare but serious skin reactions

Abbreviation: APD, Antipsychotic drug. ^1^ Source: [14,15,16].

**Table 3:** Characteristics of Adults Inpatients Included in the Study (*n* = 5,539,077) ^1.^

Characteristic	Unique Patients (Counts *n*)	Proportion (%)
Sex		
Female	3,311,071	59.8
Male	2,228,006	40.2
Age (years)		
18 to 64	3,589,269	64.8
65 to 84	1,553,011	28.0
≥85	396,797	7.2
Race		
African American	835,291	15.1
Caucasian	4,177,620	75.4
Hispanic	111,769	2.0
Other	414,397	7.5
Census Region		
Midwest	1,018,853	18.4
Northeast	1,741,829	31.5
South	1,859,425	33.6
West	918,970	16.6
Hospital Status		
Rural	1,089,222	19.7
Urban	4,449,855	80.3
Teaching Status		
Teaching	3,244,633	67.8
Non-Teaching	1,538,575	32.2
Missing	755,869	13.6

^1^ Other Races include Asian, biracial, Pacific Islander, Middle Eastern, and Native American.

## Data Availability

Data used in this analysis were derived from the legacy Health Facts database originally developed by Cerner Corporation, which has since been replaced by the Oracle Learning Health Network (procedures for accessing the new database are described at www.oracle.com/life-sciences/learning-health-network/ (accessed on 9 January 2026). Annual and quarterly prevalence data on APD use are tabulated in Tables S1–S6 in Supplemental Material.

## Supplementary Materials

The following supporting information can be downloaded at: https://www.mdpi.com/article/10.3390/pharmacy14010014/s1, Table S1: Standardized annual prevalence of use of Antipsychotic Drugs by class; Table S2: Standardized Annual Prevalence of Use of individual Typical Antipsychotic Drugs in Adult Inpatients, 2000–2016, United States; Table S3: Standardized Annual Prevalence of Use of individual Atypical Antipsychotic Drugs in Adult Inpatients, 2000–2016, United States; Table S4: Standardized Quarterly Prevalence of Use of Atypical and Typical Antipsychotic Drugs by Class in Adult Inpatients, 2000–2016, United States; Table S5: Standardized Quarterly Prevalence of Use of Individual Typical Antipsychotic Drugs in Adult Inpatients, 2000–2016, United States; Table S6: Standardized Quarterly Prevalence of Use of Individual Atypical Antipsychotic Drugs in Adult Inpatients, 2000–2016, United States.

## Abbreviations

The following abbreviations are used in this manuscript:

APD	Antipsychotic Drug
D2	Dopamine D2
SD	Standard Deviation
EHR	Electronic Health Records
FDA	Food and Drug Administration
FGA	First Generation Antipsychotic
5-HT_2A_	5-hydroxytryptamine receptor 2A
SGA	Second Generation Antipsychotic
NHANES	National health and Nutrition Examination Survey
US	United States
