# Supplementary material for: Trends in Antipsychotic Drug Use in the United States, 2000–2016
Source: Pharmacy (Basel). 2026 Jan 24;14(1):14. doi: 10.3390/pharmacy14010014 (PMC12921739; doi:10.3390/pharmacy14010014)
Supplement: Supplementary file 1 [file pharmacy-14-00014-s001.zip › pharmacy-4046961-supplementary.pdf]

SUPPLEMENTAL MATERIAL

The objective of the Supplemental Material is to provide a more fulsome overview of the annual and quarterly prevalence of use of antipsychotic drugs (APDs) by class and Individual drugs in adult inpatients (≥18 years of age). This includes:

- Standardized annual prevalence of use of APDs by class (Table S1.).
- Standardized prevalence of use of atypical and typical APDs (Tables S2. and S3.)
- Standardized quarterly prevalence of use of APDs by class (Table S4.)
- Standardized quarterly prevalence of use of atypical and typical APDs (Tables S5. and S6.).

Table S1. Annual Prevalence of Use of Atypical and Typical Antipsychotic Drugs by Class, 2000 – 2016, United States.

Year	Atypical Antipsychotic Drugs	Typical Antipsychotic Drugs
2000	2.6	13.1
2001	3.7	15.0
2002	3.6	9.1
2003	4.3	8.2
2004	6.4	11.4
2005	6.4	12.5
2006	5.7	12.3

2007	5.4	10.9
2008	5.1	10.2
2009	5.5	11.9
2010	5.7	10.1
2011	5.4	11.6
2012	5.7	10.3
2013	6.1	10.0
2014	6.1	8.6
2015	6.6	8.0
2016	6.6	8.8

Table S2. Standardized Annual Prevalence of Use of individual Typical Antipsychotic Drugs in Adult Inpatients, 2000 – 2016, United States.

Year	Chlorpromazine	Droperidol	Fluphenazine	Haloperidol	Loxapine	Molindone	Perphenazine	Pimozide	Prochlorperazine	Thioridazine	Thiothixene	Trifluoperazine
2000	0.5	3.27	0.11	2.85	0.06	0	0.08	0.01	7.08	0.11	0.05	0.03
2001	0.47	5.11	0.15	3.16	0.03	0	0.1	0	6.87	0.09	0.06	0.05
2002	0.34	1.39	0.09	2.8	0.03	0	0.06	0	4.78	0.05	0.03	0.02
2003	0.3	1.25	0.11	2.23	0.03	0	0.06	0	4.55	0.03	0.04	0.03
2004	0.39	1.57	0.22	2.74	0.02	0.01	0.05	0	6.65	0.04	0.03	0.03
2005	0.43	1.54	0.15	3.42	0.08	0.01	0.07	0	7.25	0.03	0.05	0.02
2006	0.37	0.87	0.14	3.51	0.02	0	0.1	0	7.7	0.02	0.03	0.03
2007	0.42	0.98	0.1	3.5	0.01	0	0.07	0	6.18	0.02	0.04	0.02
2008	0.4	1.39	0.1	3.06	0.02	0	0.08	0	5.5	0.02	0.03	0.02
2009	0.4	1.92	0.09	3	0.02	0	0.08	0	6.77	0.01	0.03	0.02
2010	0.41	1.33	0.1	2.93	0.02	0	0.07	0	5.57	0.01	0.04	0.01
2011	0.39	1.95	0.09	3.41	0.02	0	0.09	0	6.33	0.01	0.04	0.02
2012	0.4	3.2	0.1	3.95	0.02	0	0.08	0	3.38	0.01	0.05	0.01
2013	0.37	2.66	0.1	4.2	0.04	0	0.07	0	3.64	0.01	0.04	0.02
2014	0.35	1.26	0.11	3.95	0.03	0	0.05	0	3.72	0.01	0.02	0.02
2015	0.34	0.48	0.11	4.26	0.03	0	0.05	0	3.11	0.01	0.02	0.01
2016	0.37	0.33	0.09	4.69	0.02	0	0.05	0	3.61	0.01	0.01	0.01

Table S3. Standardized Annual Prevalence of Use of individual Atypical Antipsychotic Drugs in Adult Inpatients, 2000 – 2016, United States.

Year	Aripiprazole	Asenapine	Brexipiprazole	Caripiprazine	Clozapine	Iloperidone	Lurasidone	Olanzapine	Paliperidone	Pimavanserin	Quetiapine	Risperidone	Ziprasidone
2000	0	0	0	0	0.12	0	0	1.13	0	0	0.5	1.18	0
2001	0	0	0	0	0.16	0	0	1.48	0	0	1	1.44	0.12
2002	0	0	0	0	0.1	0	0	1.46	0	0	1.06	1.27	0.2
2003	0.17	0	0	0	0.1	0	0	1.43	0	0	1.68	1.28	0.47
2004	0.38	0	0	0	0.14	0	0	1.71	0	0	2.75	1.7	1.05
2005	0.62	0	0	0	0.13	0	0	1.28	0	0	3.14	1.54	1.29
2006	0.62	0	0	0	0.12	0	0	1.23	0	0	2.77	1.37	1.04
2007	0.63	0	0	0	0.12	0	0	0.97	0.02	0	2.54	1.22	0.97
2008	0.75	0	0	0	0.08	0	0	0.87	0.1	0	2.52	1.13	0.67
2009	0.98	0	0	0	0.11	0	0	0.91	0.06	0	2.65	1.23	0.73
2010	1.04	0.02	0	0	0.11	0	0	0.92	0.1	0	2.51	1.36	0.76
2011	1	0.04	0	0	0.11	0	0.01	0.97	0.13	0	2.23	1.39	0.69
2012	0.99	0.06	0	0	0.11	0.01	0.04	1.1	0.11	0	2.39	1.53	0.65
2013	0.99	0.06	0	0	0.1	0	0.09	1.49	0.12	0	2.56	1.58	0.59
2014	0.93	0.05	0	0	0.1	0.01	0.17	1.48	0.12	0	2.59	1.41	0.56
2015	0.97	0.08	0	0	0.12	0	0.23	1.84	0.19	0	2.79	1.38	0.57
2016	0.91	0.07	0	0	0.11	0	0.23	1.77	0.19	0	2.94	1.33	0.63

Table S4. Standardized Quarterly Prevalence of Use of Atypical and Typical Antipsychotic Drugs by Class in Adult Inpatients, 2000 – 2016, United States.

Quarter	Atypical Antipsychotic Drugs	Typical Antipsychotic Drugs
2000-Q1	2.25	11.27
2000-Q2	2.63	13.42
2000-Q3	2.52	13.27
2000-Q4	2.77	13.26
2001-Q1	3.51	14.23
2001-Q2	3.78	14.51
2001-Q3	3.55	16.34
2001-Q4	3.74	15
2002-Q1	3.74	12.08
2002-Q2	3.65	8.66
2002-Q3	3.53	7.73
2002-Q4	3.47	8.09
2003-Q1	3.61	7.96
2003-Q2	4.12	8.2
2003-Q3	5.02	8.43
2003-Q4	5.01	8.48
2004-Q1	5.77	9.26
2004-Q2	5.91	9.02
2004-Q3	5.98	11.53
2004-Q4	7.36	13.98
2005-Q1	7.35	12.12
2005-Q2	6.38	12.2
2005-Q3	6.06	12.78
2005-Q4	6.02	12.95
2006-Q1	5.97	12.07
2006-Q2	5.74	12.93
2006-Q3	5.61	12.35
2006-Q4	5.66	11.76
2007-Q1	5.27	10.92
2007-Q2	5.59	10.34
2007-Q3	5.15	10.33
2007-Q4	5.61	11.69
2008-Q1	5.41	10.12
2008-Q2	5.24	10.67
2008-Q3	4.99	10.27
2008-Q4	4.81	9.82

Table S4. Standardized Quarterly Prevalence of Use of Atypical and Typical Antipsychotic Drugs by Class in Adult Inpatients, 2000 – 2016, United States (cont.).

Quarter	Atypical Antipsychotic Drugs	Typical Antipsychotic Drugs
2009-Q1	5.41	11.29
2009-Q2	5.68	12.52
2009-Q3	5.62	12.48
2009-Q4	5.41	11.33
2010-Q1	5.87	10.27
2010-Q2	5.7	10.37
2010-Q3	5.78	9.68
2010-Q4	5.43	9.83
2011-Q1	5.32	11.56
2011-Q2	5.32	11.94
2011-Q3	5.7	11.83
2011-Q4	5.18	11.11
2012-Q1	5.52	11.09
2012-Q2	5.43	10.16
2012-Q3	5.88	9.51
2012-Q4	5.84	10.27
2013-Q1	5.95	9.91
2013-Q2	6.2	10.5
2013-Q3	6.23	10.27
2013-Q4	5.91	9.22
2014-Q1	6.05	8.79
2014-Q2	6.06	8.32
2014-Q3	6.11	8.61
2014-Q4	6.15	8.76
2015-Q1	6.61	8.4
2015-Q2	6.46	7.81
2015-Q3	6.82	8
2015-Q4	6.45	7.92
2016-Q1	6.67	8.04
2016-Q2	6.76	8.76
2016-Q3	6.66	8.61
2016-Q4	6.41	9.64

Table S5. Standardized Quarterly Prevalence of Use of Individual Typical Antipsychotic Drugs in Adult Inpatients, 2000 – 2016, United States.

Quarter	Chlorpromazine	Droperidol	Fluphenazine	Haloperidol	Loxapine	Molindone	Perphenazine	Pimozide	Prochlorperazine	Thioridazine	Thiothixene	Trifluoperazine
2000-Q1	0.64	2.05	0.07	3.2	0	0	0.05	0	5.93	0.06	0.03	0.06
2000-Q2	0.51	3.42	0.13	3.21	0.09	0	0.11	0.01	6.83	0.19	0.06	0.02
2000-Q3	0.46	3.01	0.12	2.65	0.07	0.01	0.07	0.01	7.79	0.11	0.07	0.02
2000-Q4	0.49	3.76	0.09	2.6	0.03	0	0.07	0	7.03	0.06	0.04	0.04
2001-Q1	0.53	4.11	0.21	3.14	0.02	0.01	0.11	0	6.94	0.12	0.06	0.04
2001-Q2	0.42	4.77	0.17	3.06	0.02	0	0.12	0	6.89	0.09	0.06	0.07
2001-Q3	0.51	5.91	0.1	3.41	0.04	0	0.08	0	7.42	0.07	0.05	0.05
2001-Q4	0.43	5.98	0.08	3.07	0.02	0	0.09	0	6.19	0.07	0.05	0.04
2002-Q1	0.47	3.49	0.1	3.13	0.04	0	0.05	0	5.53	0.07	0.04	0.03
2002-Q2	0.38	0.58	0.08	2.88	0.02	0	0.06	0	4.92	0.03	0.05	0.03
2002-Q3	0.24	0.77	0.1	2.54	0.04	0	0.05	0	4.28	0.04	0.02	0.02
2002-Q4	0.3	0.7	0.09	2.69	0.02	0	0.09	0	4.46	0.03	0.01	0.01
2003-Q1	0.32	0.96	0.11	2.63	0.01	0	0.03	0	4.24	0.02	0.04	0.02
2003-Q2	0.31	1.34	0.09	2.15	0.04	0	0.07	0	4.52	0.04	0.05	0.03
2003-Q3	0.3	1.54	0.16	1.9	0.04	0	0.09	0	4.68	0.05	0.05	0.06
2003-Q4	0.27	1.36	0.08	1.95	0.05	0.02	0.07	0	5.01	0.03	0.03	0.01
2004-Q1	0.39	0.97	0.29	2.46	0	0	0.08	0	5.33	0.05	0.02	0.03
2004-Q2	0.39	0.2	0.17	2.65	0.02	0	0.06	0.01	5.72	0.03	0.03	0.04
2004-Q3	0.34	1.33	0.25	2.45	0.02	0	0.05	0	7.52	0.02	0.02	0.01
2004-Q4	0.41	2.97	0.19	3.16	0.04	0.02	0.04	0	7.43	0.06	0.05	0.04
2005-Q1	0.39	1.59	0.19	3.27	0.05	0.01	0.08	0	6.93	0.01	0.03	0.04
2005-Q2	0.45	1.49	0.21	3.27	0.19	0	0.07	0	6.89	0.03	0.07	0.02
2005-Q3	0.45	1.69	0.09	3.4	0.05	0	0.06	0	7.45	0.05	0.05	0.01
2005-Q4	0.42	1.39	0.11	3.71	0.02	0.01	0.06	0	7.71	0.04	0.06	0.03
2006-Q1	0.41	1.48	0.15	3.46	0.02	0	0.1	0.01	6.83	0.01	0.03	0.03
2006-Q2	0.36	0.87	0.13	3.65	0.03	0	0.08	0	8.32	0.01	0.03	0.02
2006-Q3	0.34	0.66	0.13	3.54	0.01	0	0.09	0	8.05	0.03	0.03	0.03
2006-Q4	0.37	0.63	0.14	3.42	0.02	0	0.12	0.01	7.53	0.02	0.02	0.03

Table S5. Standardized Quarterly Prevalence of Use of Individual Typical Antipsychotic Drugs in Adult Inpatients, 2000 – 2016, United States (cont.).

Quarter	Chlorpromazine	Droperidol	Fluphenazine	Haloperidol	Loxapine	Molindone	Perphenazine	Pimozide	Prochlorperazine	Thioridazine	Thiothixene	Trifluoperazine
2007-Q1	0.42	0.64	0.09	3.07	0.01	0.01	0.06	0	7.01	0.02	0.03	0.01
2007-Q2	0.38	0.73	0.09	3.51	0.02	0	0.08	0	5.97	0.02	0.04	0.02
2007-Q3	0.4	0.91	0.11	3.67	0	0	0.07	0	5.57	0.03	0.03	0.02
2007-Q4	0.45	1.51	0.11	3.64	0.02	0	0.05	0	6.3	0.03	0.07	0.02
2008-Q1	0.42	1.4	0.13	3.31	0.02	0	0.09	0	5.14	0.02	0.04	0.02
2008-Q2	0.44	1.34	0.11	3.42	0.02	0	0.07	0	5.62	0.02	0.04	0.02
2008-Q3	0.36	1.43	0.08	3.01	0.01	0	0.08	0	5.61	0.02	0.03	0.02
2008-Q4	0.38	1.37	0.08	2.62	0.02	0	0.08	0	5.58	0.02	0.02	0.02
2009-Q1	0.43	1.69	0.08	2.96	0.01	0	0.08	0	6.37	0.02	0.04	0.02
2009-Q2	0.42	2.04	0.09	3.03	0.03	0	0.06	0	7.24	0.01	0.04	0.03
2009-Q3	0.36	2.12	0.09	3.12	0.02	0	0.09	0	7.04	0.01	0.02	0.01
2009-Q4	0.41	1.84	0.09	2.87	0.01	0	0.07	0	6.43	0.01	0.03	0.02
2010-Q1	0.44	1.44	0.1	2.99	0.02	0	0.07	0	5.6	0.01	0.06	0.01
2010-Q2	0.44	1.52	0.1	3.14	0.03	0	0.06	0	5.47	0.01	0.03	0.01
2010-Q3	0.36	1.14	0.09	2.86	0	0	0.08	0	5.46	0.02	0.04	0.01
2010-Q4	0.38	1.18	0.1	2.67	0.01	0	0.07	0	5.78	0.01	0.04	0.02
2011-Q1	0.45	1.52	0.07	2.96	0.03	0	0.06	0	6.9	0.01	0.03	0.01
2011-Q2	0.37	1.52	0.08	3.37	0.02	0	0.09	0	6.94	0.01	0.03	0.02
2011-Q3	0.41	1.81	0.11	3.78	0.02	0	0.11	0	6.3	0.02	0.06	0.02
2011-Q4	0.35	2.84	0.09	3.47	0.02	0	0.09	0	5.3	0.01	0.05	0.02
2012-Q1	0.36	3.12	0.1	3.7	0.02	0	0.09	0	4.65	0.02	0.04	0.02
2012-Q2	0.41	2.85	0.1	4.1	0.02	0	0.08	0	3.24	0.01	0.05	0.01
2012-Q3	0.4	3.04	0.09	3.86	0.02	0	0.09	0	2.86	0.01	0.05	0.01
2012-Q4	0.42	3.74	0.1	4.12	0.03	0	0.09	0	2.78	0	0.06	0.02
2013-Q1	0.4	3.27	0.1	4.16	0.03	0	0.1	0	2.77	0.01	0.04	0.02
2013-Q2	0.38	2.94	0.11	4.61	0.03	0	0.08	0	3.64	0.02	0.04	0.02
2013-Q3	0.4	2.56	0.09	4.31	0.05	0	0.07	0	3.97	0.01	0.03	0.02
2013-Q4	0.3	2.04	0.09	3.8	0.03	0	0.05	0	3.98	0.01	0.04	0.01

Table S5. Standardized Quarterly Prevalence of Use of Individual Typical Antipsychotic Drugs in Adult Inpatients, 2000 – 2016, United States (cont.).

Quarter	Chlorpromazine	Droperidol	Fluphenazine	Haloperidol	Loxapine	Molindone	Perphenazine	Pimozide	Prochlorperazine	Thioridazine	Thiothixene	Trifluoperazine
2014-Q1	0.33	1.57	0.14	3.95	0.03	0	0.05	0	3.67	0.01	0.02	0.03
2014-Q2	0.32	1.4	0.11	3.89	0.02	0	0.05	0	3.48	0.01	0.01	0.01
2014-Q3	0.37	1.33	0.09	3.95	0.02	0	0.05	0	3.75	0.01	0.02	0.01
2014-Q4	0.38	0.76	0.11	4	0.03	0	0.04	0	3.96	0.01	0.02	0.01
2015-Q1	0.38	0.55	0.12	4.24	0.04	0	0.05	0	3.46	0.01	0.02	0.01
2015-Q2	0.35	0.53	0.1	4.28	0.03	0	0.05	0	2.8	0.01	0.02	0.01
2015-Q3	0.3	0.46	0.12	4.2	0.04	0	0.05	0	3.14	0.01	0.02	0.01
2015-Q4	0.34	0.36	0.11	4.33	0.02	0	0.06	0	3.01	0.01	0.02	0.01
2016-Q1	0.38	0.37	0.11	4.61	0.01	0	0.06	0	2.85	0.01	0.01	0.01
2016-Q2	0.36	0.34	0.11	4.85	0.02	0	0.05	0	3.42	0.01	0.01	0
2016-Q3	0.4	0.29	0.08	4.54	0.03	0	0.05	0	3.56	0.01	0.01	0.01
2016-Q4	0.35	0.33	0.07	4.77	0.02	0	0.04	0	4.51	0.01	0.01	0.01

Table S6. Standardized Quarterly Prevalence of Use of Individual Atypical Antipsychotic Drugs in Adult Inpatients, 2000 – 2016, United States.

Quarter	Aripiprazole	Asenapine	Brexipiprazole	Caripiprazine	Clozapine	Iloperidone	Lurasidone	Olanzapine	Paliperidone	Pimavanserin	Quetiapine	Risperidone	Ziprasidone
2000-Q1	0	0	0	0	0.02	0	0	0.9	0	0	0.37	1.18	0
2000-Q2	0	0	0	0	0.18	0	0	1.13	0	0	0.43	1.28	0
2000-Q3	0	0	0	0	0.11	0	0	1.13	0	0	0.42	1.14	0
2000-Q4	0	0	0	0	0.09	0	0	1.2	0	0	0.67	1.13	0
2001-Q1	0	0	0	0	0.16	0	0	1.44	0	0	1	1.43	0.01
2001-Q2	0	0	0	0	0.19	0	0	1.57	0	0	1.01	1.48	0.11
2001-Q3	0	0	0	0	0.19	0	0	1.3	0	0	0.92	1.46	0.19
2001-Q4	0	0	0	0	0.08	0	0	1.58	0	0	1.07	1.37	0.19
2002-Q1	0	0	0	0	0.1	0	0	1.63	0	0	1	1.33	0.15
2002-Q2	0	0	0	0	0.07	0	0	1.47	0	0	1.02	1.38	0.2
2002-Q3	0	0	0	0	0.07	0	0	1.32	0	0	1.12	1.25	0.22
2002-Q4	0	0	0	0	0.16	0	0	1.43	0	0	1.07	1.14	0.23
2003-Q1	0.04	0	0	0	0.1	0	0	1.21	0	0	1.3	1.21	0.26
2003-Q2	0.16	0	0	0	0.1	0	0	1.31	0	0	1.72	1.24	0.38
2003-Q3	0.29	0	0	0	0.11	0	0	1.57	0	0	2.08	1.44	0.76
2003-Q4	0.29	0	0	0	0.12	0	0	1.88	0	0	1.93	1.3	0.68
2004-Q1	0.25	0	0	0	0.16	0	0	2	0	0	2.18	1.49	0.73
2004-Q2	0.27	0	0	0	0.13	0	0	1.85	0	0	2.57	1.61	0.85
2004-Q3	0.31	0	0	0	0.13	0	0	1.54	0	0	2.66	1.68	0.91
2004-Q4	0.58	0	0	0	0.13	0	0	1.57	0	0	3.29	1.88	1.48
2005-Q1	0.61	0	0	0	0.1	0	0	1.65	0	0	3.38	1.81	1.41
2005-Q2	0.57	0	0	0	0.16	0	0	1.31	0	0	3.19	1.49	1.26
2005-Q3	0.56	0	0	0	0.12	0	0	1.13	0	0	3.02	1.46	1.26
2005-Q4	0.75	0	0	0	0.12	0	0	1.05	0	0	3.02	1.42	1.26

Table S6. Standardized Quarterly Prevalence of Use of Individual Atypical Antipsychotic Drugs in Adult Inpatients, 2000 – 2016, United States (cont.).

Quarter	Aripiprazole	Asenapine	Brexipiprazole	Caripiprazine	Clozapine	Iloperidone	Lurasidone	Olanzapine	Paliperidone	Pimavanserin	Quetiapine	Risperidone	Ziprasidone
2006-Q1	0.66	0	0	0	0.13	0	0	1.16	0	0	3.1	1.36	1.1
2006-Q2	0.61	0	0	0	0.1	0	0	1.02	0	0	2.98	1.43	1.07
2006-Q3	0.62	0	0	0	0.1	0	0	1.28	0	0	2.61	1.37	1.01
2006-Q4	0.6	0	0	0	0.14	0	0	1.41	0	0	2.51	1.33	1.01
2007-Q1	0.5	0	0	0	0.14	0	0	1.05	0	0	2.53	1.17	0.95
2007-Q2	0.62	0	0	0	0.12	0	0	1.06	0	0	2.72	1.17	1.09
2007-Q3	0.6	0	0	0	0.1	0	0	0.88	0.02	0	2.35	1.19	0.9
2007-Q4	0.76	0	0	0	0.12	0	0	0.93	0.04	0	2.54	1.31	0.94
2008-Q1	0.69	0	0	0	0.07	0	0	0.97	0.1	0	2.68	1.29	0.72
2008-Q2	0.77	0	0	0	0.09	0	0	0.94	0.11	0	2.61	1.13	0.72
2008-Q3	0.74	0	0	0	0.1	0	0	0.8	0.1	0	2.49	1.1	0.62
2008-Q4	0.8	0	0	0	0.06	0	0	0.81	0.08	0	2.33	1.04	0.62
2009-Q1	0.95	0	0	0	0.11	0	0	0.77	0.08	0	2.63	1.25	0.75
2009-Q2	1	0	0	0	0.11	0	0	0.93	0.07	0	2.74	1.29	0.75
2009-Q3	0.96	0	0	0	0.12	0	0	0.96	0.04	0	2.69	1.23	0.7
2009-Q4	0.99	0	0	0	0.11	0	0	0.95	0.05	0	2.55	1.15	0.71
2010-Q1	1.06	0.02	0	0	0.11	0	0	0.95	0.09	0	2.6	1.34	0.82
2010-Q2	1.06	0.03	0	0	0.11	0	0	0.88	0.09	0	2.56	1.35	0.76
2010-Q3	1.04	0.02	0	0	0.11	0.01	0	0.91	0.11	0	2.52	1.45	0.71
2010-Q4	1	0.01	0	0	0.12	0.01	0	0.93	0.13	0	2.31	1.29	0.76
2011-Q1	0.99	0.02	0	0	0.1	0	0	0.93	0.11	0	2.35	1.2	0.71
2011-Q2	1.06	0.04	0	0	0.1	0	0	0.92	0.12	0	2.22	1.26	0.69
2011-Q3	1.05	0.07	0	0	0.14	0	0.01	1.01	0.15	0	2.29	1.58	0.73
2011-Q4	0.9	0.04	0	0	0.09	0	0.02	1.01	0.14	0	2.1	1.47	0.62

Table S6. Standardized Quarterly Prevalence of Use of Individual Atypical Antipsychotic Drugs in Adult Inpatients, 2000 – 2016, US (cont.).

Quarter	Aripiprazole	Asenapine	Brexipiprazole	Caripiprazine	Clozapine	Iloperidone	Lurasidone	Olanzapine	Paliperidone	Pimavanserin	Quetiapine	Risperidone	Ziprasidone
2012-Q1	0.97	0.03	0	0	0.1	0.01	0.01	0.98	0.11	0	2.29	1.64	0.64
2012-Q2	0.99	0.06	0	0	0.13	0.01	0.03	1	0.14	0	2.19	1.43	0.67
2012-Q3	1.01	0.07	0	0	0.11	0	0.06	1.2	0.09	0	2.51	1.58	0.6
2012-Q4	0.99	0.08	0	0	0.12	0.01	0.05	1.2	0.11	0	2.55	1.49	0.67
2013-Q1	0.99	0.04	0	0	0.12	0	0.06	1.45	0.13	0	2.46	1.58	0.6
2013-Q2	1.04	0.07	0	0	0.1	0	0.07	1.43	0.13	0	2.57	1.67	0.62
2013-Q3	1.01	0.06	0	0	0.09	0	0.1	1.66	0.13	0	2.59	1.57	0.58
2013-Q4	0.93	0.06	0	0	0.09	0	0.1	1.42	0.11	0	2.58	1.51	0.56
2014-Q1	0.89	0.02	0	0	0.1	0.01	0.12	1.45	0.13	0	2.56	1.52	0.55
2014-Q2	0.91	0.04	0	0	0.09	0.01	0.17	1.48	0.09	0	2.58	1.45	0.56
2014-Q3	0.94	0.06	0	0	0.1	0.01	0.19	1.44	0.12	0	2.65	1.32	0.56
2014-Q4	0.97	0.07	0	0	0.09	0	0.2	1.55	0.13	0	2.57	1.37	0.57
2015-Q1	0.97	0.05	0	0	0.12	0	0.21	1.76	0.17	0	2.81	1.44	0.53
2015-Q2	1.01	0.09	0	0	0.13	0	0.22	1.76	0.18	0	2.69	1.38	0.54
2015-Q3	0.98	0.08	0	0	0.11	0	0.26	2.05	0.21	0	2.82	1.36	0.6
2015-Q4	0.91	0.1	0	0	0.1	0	0.22	1.81	0.18	0	2.86	1.35	0.59
2016-Q1	0.93	0.05	0	0	0.12	0	0.22	1.8	0.16	0	2.94	1.38	0.58
2016-Q2	0.93	0.07	0	0	0.1	0	0.24	1.92	0.22	0	2.87	1.36	0.65
2016-Q3	0.89	0.07	0	0	0.12	0	0.23	1.78	0.2	0	2.96	1.33	0.7
2016-Q4	0.88	0.06	0	0	0.11	0	0.25	1.59	0.17	0	2.97	1.24	0.59
